# Unusual tumour ablations: report of difficult and interesting cases

**DOI:** 10.3332/ecancer.2017.733

**Published:** 2017-04-18

**Authors:** Giovanni Mauri, Luca Nicosia, Gianluca Maria Varano, Paul Shyn, Sergio Sartori, Paola Tombesi, Francesca Di Vece, Franco Orsi, Luigi Solbiati

**Affiliations:** 1 Division of Interventional Radiology, European Institute of Oncology, Milan, Italy; 2 Postgraduate School of Radiology, University of Milan, Italy; 3 Abdominal Imaging and Intervention, Department of Radiology, Brigham and Women’s Hospital, Harvard Medical School, Boston, MA, USA; 4 Section of Interventional Ultrasound, Department of Internal Medicine, St Anna Hospital Ferrara, Italy; 5 Department of Radiology, Humanitas University and Research Hospital, Rozzano (Milan), Italy

**Keywords:** image-guided ablation, thermal ablation, protective manoeuver, complex cases, radiofrequency ablation, laser ablation, microwave ablation, cryoablation, contrast-enhanced ultrasound, PET/CT

## Abstract

Image-guided ablations are nowadays applied in the treatment of a wide group of diseases and in different organs and regions, and every day interventional radiologists have to face more difficult and unusual cases of tumour ablation. In the present case review, we report four difficult and unusual cases, reporting some tips and tricks for a successful image-guided treatment.

## Introduction

In recent years, interventional radiology has had a rapidly increasing diffusion worldwide, being recognised as the therapeutic modality of choice in several fields: vascular and biliary procedures, management of post-surgical complications and treatment of cancer patients [1–6]. In the field of oncology, interventional radiology is nowadays recognised as the ‘fourth pillar’ together with medical oncology, surgical oncology, and radiotherapy. This has been made possible mainly because of the continuous technical improvements in image-guided procedures (biopsies, thermal ablations, etc). The increasing evidence emerging from the literature about the safety and effectiveness of these techniques is prompting their application in a wider group of diseases and in different organs and regions, more recently including ‘unusual’ body regions, such as lung and neck [7–9]. With the wider and wider diffusion of image-guided therapies, and the recognition of the clinical value of such minimally invasive treatments by referring physicians, a larger number of complex cases are coming to the attention of interventional radiologists. Thus, the knowledge of the most advanced techniques to deal with difficult cases is now becoming crucial to offer highly effective treatments to a larger patients’ population. Moreover, it is important for the best care of oncological patients that the referring physicians and oncologists are aware of the different treatment options that interventional radiologists can offer, nowadays even in unusual and difficult cases.

In the present case review, some difficult and unusual cases, treated using tips and tricks, are presented and discussed.

### CASE 1: PET only visible liver metastasis treated with fusion image guidance

**Figure 1.**
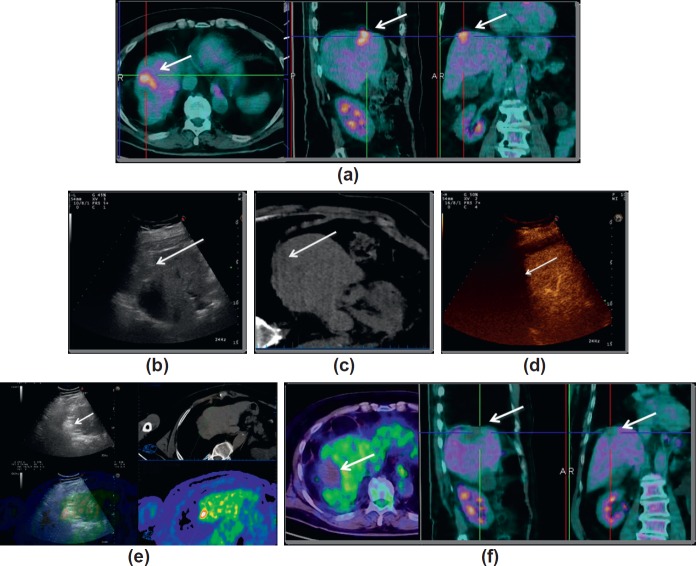
Case of a patient previously treated for colorectal cancer presenting with PET-positive metastasis that was treated with percutaneous microwave ablation guided by fusion imaging and virtual navigation. (a). PET/CT images demonstrating a 4 cm FDG avid recurrence (arrow) located at the liver dome. (b). The lesion was completely invisible at ultrasound (arrow). (c). The lesion was undetectable at non-enhanced CT too (arrow). (d). Even contrast-enhanced ultrasound was not able to show the lesion due to the presence of interposed aerated lung parenchyma (arrow). (e). The treatment was performed using a system for image fusion that shows the real-time ultrasound in the upper left quadrant (arrow gas forming during ablation), the CT images on the upper right quadrant, the PET images in the lower right quadrant and the fused images in the lower left quadrant. (f). PET/CT after treatment showing complete ablation of the treated lesion.

A 58-year-old male with history of resection of colorectal cancer two years earlier and percutaneous image-guided thermal ablation of a hepatic metastasis at segment VIII 10 months earlier was referred to our institution for possible local treatment of a 4 cm recurrence on the cranial side of the previously ablated area, which was detected by 18-FDG-PET/CT (Figure 1a). Given that in our centre, most liver thermal ablations are performed under ultrasound (US) guidance, we preliminarily performed an US examination to localise the lesion, but unfortunately, it was completely invisible on B-mode US (Figure 1b) due to its location in the liver dome, completely hidden behind the aerated lung. It was undetectable also on the unenhanced computed tomography (CT) of the positron emission tomography-CT (PET-CT) study (Figure 1c). In cases of poor sonographic visibility, contrast-enhanced ultrasound (CEUS) with the administration of dedicated contrast agent (microbubbles) has been reported to be extremely helpful to enhance the conspicuity of a lesion and to enhance the accuracy of image-guided targeting [10, 11]. Thus, we attempted to enhance the visibility of the lesion with CEUS, but due to the presence of the aerated lung, it was impossible to detect the lesion (Figure 1d). At this stage, only PET-CT could have been directly used to guide the ablation. The guidance of ablations by PET/CT-guided has been reported to be feasible and effective [12, 13] but a dedicated PET/CT room, and an experienced operator are needed for such a treatment. An interventional PET/CT room was not available, and thus, we moved to a different solution. For lesions invisible with US and not completely depictable with CEUS, virtual navigation systems and fusion imaging (fusion of datasets produced by different imaging modalities) have been reported to be a safe, feasible, and effective strategy to guide percutaneous ablations [14–16]. With an ultrasound machine equipped with dedicated software and hardware, a magnetic field generator and a series of sensors applied to US probe and ablative device, a precise coregistration of previously acquired volumetric datasets (from CT, MRI, or PET/CT) and real-time US can be performed in the US interventional room. This was the method used to treat the patient. With the patient under general anaesthesia, the PET/CT volumetric data set imported into the US machine was coregistered with real-time US using 1–2 internal markers, such as vessel bifurcations. This step may require a few minutes depending on the operator’s experience and skill, but new systems for partially automatic coregistration are making this procedure shorter and easier [17, 18]. Moreover, if an additional sensor is attached to the ablative device, the system is able to show a virtual needle projected on the expected path of the ablative device, thus enabling to reach the target even in cases of poor US visibility [19, 20]. In this patient, we decided to perform the ablation using a microwave antenna. With a single antenna insertion, microwaves allow the achievement of larger ablative areas, in shorter time and with significantly less heat-sink effect compared to the other ablative modalities [21–24]. During ablation, the gas produced by heating was used to monitor the correctness of targeting (Figure 1e). At the end, we performed CEUS in order to better highlight the margins of the ablated area. Even if it was not possible to precisely evaluate the most cranial part of the treated area, the avascular area was considered to completely encompass that of uptake showed on PET/CT and the procedure was considered completed. CEUS performed immediately after the treatment allows to visualise incomplete ablations and to guide a subsequent ablation into the still viable tumour in the same session [10, 25–27], thus decreasing the number of second ablative sessions, improving clinical results and reducing costs. At 24 hours from the treatment, we performed a PET/CT examinations, which demonstrated the complete ablation of the metastasis (Figure 1f).

### CASE 2. CT-guided microwave ablation of an adrenal mass

**Figure 2.**
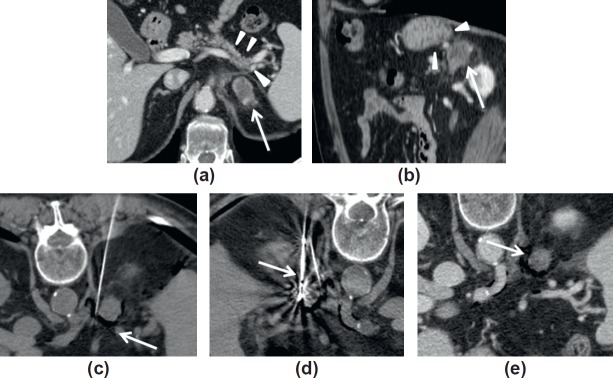
Case of an adrenal metastasis from lung cancer treated with percutaneous microwave ablation. (a,b). Preoperative CT demonstrated a 31 mm right adrenal metastasis (arrow) that was located in close proximity with critical structures such as pancreas (arrowheads in a) or stomach (arrowheads in b). (c). A small caliber needle was placed medially to the adrenal gland and CO_2_ injected in order to displace the lesion from surrounding critical structures. (d). A microwave antenna was inserted into the adrenal metastasis (arrow) and ablation was performed. (e). Final result 24 hours after ablation showing complete devascularisation of the lesion (arrow).

A 58-year-old male was referred to us for the evaluation of a possible percutaneous treatment for an adrenal metastasis from a previously resected lung cancer. The patient underwent 4 years before right upper lung lobe resection for a high-grade lung papillary adenocarcinoma. One year later, he developed a new lung lesion in the right lower lobe, and brain and left adrenal metastases, treated with repeated sessions of radiation therapy. The treatments achieved a good disease control at the brain level, meanwhile the adrenal lesion relapsed. The patient was referred to our Department of Interventional Radiology for local ablative treatment as radiation therapy was considered no more feasible. When the patient was evaluated for ablation, the adrenal lesion measured 31 mm (Figure 2a). The patient was considered suitable for a CT-guided microwave ablation. Percutaneous image-guided ablations have been reported to be feasible for the treatment of adrenal gland metastases [28–30].

Under general anaesthesia, the patient was placed in the prone position. As the adrenal lesion was very close to sensitive surrounding structures, such as the stomach, the colon and the pancreas (Figure 2a, 2b), percutaneous injection of carbon dioxide (CO_2_) was planned in order to displace the sensible structure from the adrenal gland. A protective manoeuver, such as the injection of fluid, gas or dedicated gel, has been successfully reported in order to displace the surrounding structure from the target of an image-guided ablation [30–32]. These techniques may increase the safety of image-guided procedures and help to enrol patients otherwise unsuitable for percutaneous treatment due to the high risk of complications. A 21 G needle was percutaneously placed under CT guidance medially and deeper to the lesion, in its close proximity (Figure 2c). Once the needle was in the planned position, a 20-ml syringe was filled with CO_2_, and manual gas injection was performed. CO_2_ is particularly helpful in some cases, as it displaces the surrounding structures and also allows the achievement of a thermal insulation between the sensitive structures and of the area to be treated, as, compared to fluid materials, gas conduces by far less the heating. On the contrary, gas is compressible and easily diffuses into the soft tissues following the less resistance plane and may not be sufficient to achieve the planned displacement. Thus, it is important to exactly understand where to place the small needle in order to reach the desired displacement. In our case, we planned to place the needle as close as possible to the adrenal gland, with the aim of achieving a gas distribution exactly around the gland. After the injection of CO_2_, we were able to achieve a good distribution of the gas around the gland and a good displacement of the surrounding structures (Figure 2c). At this point, the microwave antenna was inserted into the gland and the ablation was performed by using an energy of 60W for 6 min (Figure 2d). Microwaves represent a fast spreading technique for percutaneous ablation, as it allows the delivery of high energy in quite a short time, thus offering the possibility of achieving larger ablation volumes in a shorter time if compared to other techniques, such as radiofrequency [21, 22]. No complications occurred after the treatment and the patient had a regular clinical course and was discharged the day after treatment. The control performed with contrast enhanced CT 24 hours after the treatment demonstrated the complete devascularisation of the treated lesion (Figure 2e), which was considered to be completely ablated.

### CASE 3: CEUS-guided laser liver ablation

**Figure 3.**
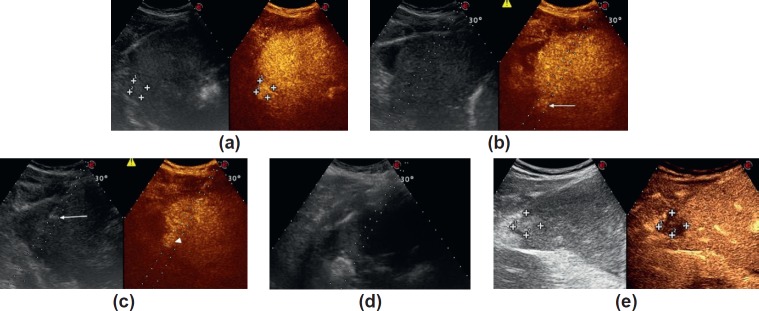
Laser ablation of a small HCC with US and CEUS guidance. (a). Oblique subcostal US (left side of the split screen) and contrast-enhanced US (right side of the split screen) scan of the right lobe of the liver, showing a 12 × 11 mm in size hyper-enhancing HCC in segment VI, strictly close to the liver capsule. (b). Oblique subcostal contrast-enhanced US scan of the right lobe of the liver, showing a residual hyper-enhancing focus 5 mm in size in the posterior portion of the nodule treated with LTA (arrow, right side of the split screen). (c). CEUS-guided insertion of the needle (arrow, right side of the split screen) towards the hyper-enhancing target (arrowhead, left side of the split screen); (d). A hyperechoic cloud is produced after delivery of 1800 J in 6 minutes. (e). Oblique intercostal contrast-enhanced US scan performed 5 minutes after retreatment showing a coagulation area of 20 × 16 mm with no evidence of viable tumour.

A 49-year-old man affected by Child-Pugh C cirrhosis from hepatitis C infection was referred to our Interventional Ultrasound Unit to undergo thermal ablation of two nodules of hepatocellular carcinoma (HCC) as a bridge to transplantation. One HCC was located in segment VII and measured 36 × 28 mm, the other one was located in segment VI strictly close to the liver capsule and measured 12 × 11 mm (Figure 3a). Based on a recently proposed algorithm to tailor the choice of the thermal ablation technique according to patient and tumour characteristics [24], the former nodule underwent microwave ablation (MWA) because of its large size. The most recent improvements in MWA technology have developed 14-gauge and 16-gauge internally cooled MWA antennas provided with a miniaturised device on the tip of the antenna (‘mini-choke’®) as a remedy to back heating effects, due to reflected waves along the coaxial line. A randomised prospective comparison between this MWA system and internally cooled radiofrequency ablation (RFA) has reported significantly larger *in vivo* coagulation areas with MWA than with RFA, after a single insertion of a 16-gauge internally cooled, minichoked antenna [23].

According to the same proposed algorithm [24], laser thermal ablation (LTA) was preferred for the latter nodule, because of its small size and at-risk location. LTA, according to the technique proposed by Pacella [33] and modified by Di Costanzo [34], utilises 300 μm bare optical fibres introduced into the tumour trough 21-gauge needles. The diameter of LA needles is smaller than RFA electrodes and MWA antennas, making LTA safer and more suitable to ablate lesions with at-risk location and/or difficult to be reached. Moreover, the use of a multisource device allows the use of up to four fibres at once: one to two fibres are usually used to treat nodules up to 1.5 cm in diameter, three fibres to treat nodules from 1.5 to 2.5 cm, and four fibres with tips arranged in a square configuration to treat nodules > 2.5 cm. Because of such a peculiarity, LTA has also been proposed as the ablation technique of choice in patients with multiple and small tumours variable in size [35].

Both MWA and LTA procedures were performed under ultrasonography (US) guidance and under conscious sedation. MWA was carried out by using a generator with a frequency of 2450 MHz and a 16-G internally cooled minichoked antenna (AMICA MWA System, HS Hospital Service, Aprilia, Italy), with power output of 70 W and duration of energy delivery of 12 min. LTA was performed by using a diode laser unit (Echolaser, Elesta srl, Florence, Italy). Two 21-gauge Chiba needles spaced 12 mm each other were placed in the anterior border of the tumour, and two bare-tip, 300 μm in diameter laser fibres were introduced through the needles and advanced until the tip of the fibres was placed into the tumour 1 cm beyond the tip of the needle, and 1800 Joules per fibre was delivered in 6 min. Contrast-enhanced US (CEUS) performed about 5 min after the end of the procedures depicted a coagulation area of 44 × 32 mm in segment VII with no evidence of residual hyper-enhancing foci, suggesting complete necrosis of the nodule treated with MWA. Conversely, a viable, hyper-enhancing focus of 5 mm in diameter was observed in the posterior portion of the nodule in segment VI treated with LTA (Figure 3b). Immediate post-procedural CEUS is strongly recommended to assess the completeness of the ablation treatment and to guide the immediate re-treatment in case of residual viable tissue [25, 36, 37].

The treatment was immediately repeated, and two laser fibres spaced 6 mm next to each other were placed into the residual tumour under CEUS guidance (Figure 3c). A further 1800 Joules per fibre was delivered in 6 min (Figure 3d), and CEUS performed 5 min after the retreatment showed a coagulation area of 20 × 16 mm with no evidence of viable tumour (Figure 3e).

### CASE 4: PET/CT-guided cryoablation of perisplenic metastases in a patient with uterine cancer

A 57-year-old female was diagnosed with endometrial adenocarcinoma two years previously and was treated with total laparoscopic hysterectomy, bilateral salpingo-oophorectomy and complete pelvic and limited peri-aortic lymphadenectomy. Histopathology revealed carcinosarcoma of the uterus. The patient subsequently received chemotherapy including carboplatin and taxol. One year later, a recurrent pelvic mass was detected on CT and patient underwent pelvic tumour resection including resection of the terminal ileum and anterior rectum. This was followed by pelvic external beam radiation therapy and concurrent cisplatin.

**Figure 4.**
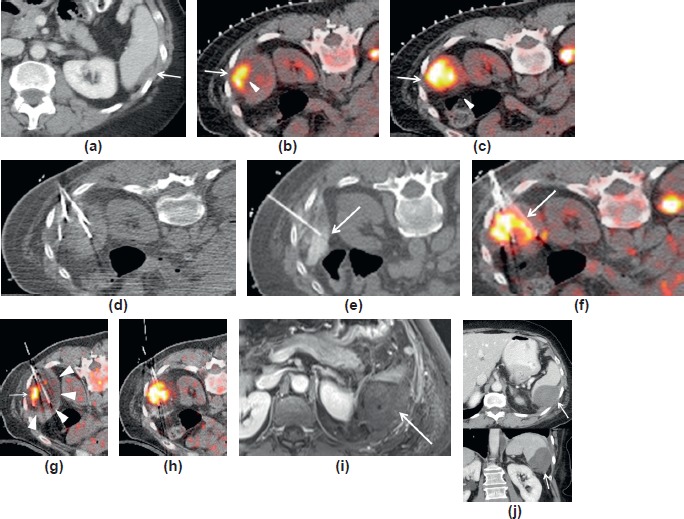
PET/CT-guided cryoablation of perisplenic metastasis in a patient with uterine cancer. (a). Contrast-enhanced CT shows the hypodense mass along the posterolateral border of the spleen (arrow) confirming a solitary peritoneal metastases. (b). Fused PET/CT image acquired using 35 s suspended ventilation prior to probe placement shows the FDG-avid perisplenic peritoneal mass positioned posterior and lateral to the spleen (arrow). The metastatic mass appears partially necrotic in the medial part of the tumour and indents the spleen (arrowheads). Note the radio-opaque skin markers overlying the spleen which are placed in order to help plan the exact coordinates for the placement of the cryoablation probes. (c). Another slice level of the fused planning PET/CT showing large FDG-avid perisplenic peritoneal metastasis (arrow) with its medial surface in close proximity to the left kidney and the anterior surface adjacent to the spenic flexure of the colon (arrowhead). (d). CT fluoroscopy images show placement of the needles and probes into the tumour. (e). CT fluoroscopy shows placement of a 20-G Chiba needle for hydrodisplacement of the splenic flexure of colon with instillation of dilute iodinated contrast into the space between tumour and colonic wall. This manoeuver prevents cryoablation injury of the bowel. (f). Fused PET/CT image shows placement of the hydrodisplacement needle between tumour and bowel wall (arrow). (g). Fused PET/CT at the level of the upper cryoablation probe performed after initial cryoablation shows a hypointense region encompassing the tumour (white arrowheads) which represents the cryoablation iceball. The area of FDG uptake (red arrow) remains unchanged as compared to initial planning PET/CT since FDG does not dissipate from the tumour during or following cryoablation. (h). Fused PET/CT performed towards the end of the cryoablation at the level of lower cryoablation probes shows persistent FDG uptake within the tumour and a hypodense iceball on CT extending beyond the FDG avid tumour. This confirms an adequate ablation margin including and extending beyond the entire tumour. Note the bowel wall is at a significant distant from the cryoablation zone which has been achieved by hydrodisplacement. (i) 3D VIBE breath hold contrast-enhanced MRI shows the hypoenhancing ablation zone (red arrow). No enhancing residual tumour visualised. (j). Follow-up contrast-enhanced CT (axial and coronal) shows the hypoenhancing ablation zone without enhancing residual tumour. There is a small area of reactive left pleural effusion (white arrow) which is expected following ablations adjacent to the diaphragm. The left renal margin adjacent to the ablation zone in the coronal image does not show significant cryoablation-related hypodensity, thus ensuring lack of significant renal parenchymal damage.

A follow-up FDG PET/CT was performed to evaluate for residual pelvic metastases and new lesions. PET/CT revealed a markedly FDG avid lesion, hypodense on CT, along the posterolateral border of the spleen suggestive of metastatic peritoneal disease (Figure 4a). Pelvic metastases were not visualised. Chemotherapy with doxorubicin was initiated, but the patient could not tolerate more than two doses.

In view of the presence of a solitary peritoneal metastasis located at the posterolateral border of spleen (Figure 4a) and considered approachable for a percutaneous ablation procedure, the patient was offered FDG PET/TC-guided percutaneous cryoablation. Percutaneous image-guided ablation can be proposed as a safe and effective treatment for limited peritoneal metastases [32, 38].

The patient was injected with 5 mCi of F18 FDG one hour prior to initiation of the procedure. The cryoablation procedure was carried out using a PET/CT scanner is equipped with CT fluoroscopy (Biograph mCT, Siemens Healthcare, Erlangen, Germany). The patient was under general anaesthesia. Cryoablation has been used for successfully treating a wide variety of tumours [28, 32, 39–42]. The patient was placed prone on the PET/CT table in order to approach the lesion from a posterior and lateral low-intercostal space. A limited planning FDG PET/CT was performed covering one bed position during a single breath-hold period of 35 s achieved by suspending ventilation during general anaesthesia. PET/CT can be effectively used to guide percutaneous biopsies and ablations [12, 43]. A single breath hold period has been reported as sufficient for good visualisation of the target lesion [12, 13]. Both the CT and PET acquisitions are obtained during this single suspended respiration. The PET acquisition time was limited to only 15 s of this 35-s PET/CT acquisition. Suspended ventilation eliminates respiratory motion of both CT and PET thereby eliminating motion-related blurring which would otherwise degrade tumour definition and ablation device depiction. The resulting fused PET/CT images are also perfectly coregistered (Figure 4b and c).

The position and size of the lesion presents several challenges. The presence of bowel so close to the anterior margin of the tumour in the prone treatment position presents a risk of injuring the bowel during cryoablation which may lead to bowel perforation. In order to avoid this complication, infusion of saline with iodinated contrast in the space between the tumour and the bowel was planned so as to displace the bowel wall away from the tumour and avoid the bowel contact with the cryoablation ice ball. Protective manoeuvres can be applied to increase the feasibility and safety of percutaneous ablations [30, 44]. The kidney which is present at the medial margin of the tumour is more tolerant of cryoablation, but care needs to be taken to avoid freezing too much renal parenchyma.

Based on the fused PET/CT images, a plan was made for the cryoablation procedure including selection of the entry point, as well as the direction and depth of cryoablation probe insertions to achieve complete ablation of the entire tumour with margins. Needle placement for the instillation of saline with contrast for bowel displacement was also determined from the planning PET/CT.

Following a small skin incision, the saline infusion needle was inserted under CT fluoroscopic guidance to reach the space between the anterior margin of the tumour and the bowel wall and a small volume of saline mixed with CT contrast was infused (Figures 4d, e and e). CT fluoroscopy was used to check the position of the instilled saline.

Following instillation of saline and CT fluoroscopic confirmation of increased separation of bowel wall from the tumour, two cryoablation probes (Ice Rods, CryoHit, Galil Medical, Yokneam, Israel) were inserted based on PET/CT-based plan into the superior and inferior parts of the tumour using fluoroscopic guidance. Following probe placement and prior to cryoablation, a single-bed position PET/CT was performed using a 35-s suspension of ventilation to acquire both CT and PET without respiratory motion related blurring or misregistration. After PET/CT confirmation of proper placement of both cryoablation probes and hydro displacement needle, the cryoablation was started (Figure 4f). PET/CT performed during the cryoablation shows growth of the hypodense iceball encompassing the entire tumour (Figure 4g and h). Iceballs formed during cryoablation can be very well detected with CT. Cryoablation was followed by thawing and re-freezing of the tumour which enhances cellular damage/death within tumour. Immediately after the initial cryoablation, another single-bed position PET/CT was performed using a suspended respiration for 35 s.

Cryoablation freezes the tumour and creates an iceball involving the tumour and adjacent tissues; the FDG within tumour remains trapped even after ablation. This helps confirm that the cryoablation iceball, represented by the hypodense zone on CT extends beyond the FDG-avid tumour, thus ensuring adequate coverage of tumour plus a safety margin with cryoablation [45, 46].

The patient was discharged the following day after an uneventful recovery. She underwent a follow-up MRI the next day to assess the results of the cryoablation and to provide a baseline for future follow-up (Figure 4i).

A follow-up CT scan after 2 weeks showed the hypo-enhancing ablation zone without enhancing residual tumour. (Figure 4l)

### Learning points

- In cases of poor visibility at US, the use of contrast-enhanced ultrasound (CEUS) with the administration of dedicated contrast agent has been reported to be extremely helpful to enhance the conspicuity of a lesion in order to better depict it and to enhance the accuracy of image-guided targeting.
- PET/CT-guided ablations have been reported to be feasible and effective.
- In cases of lesions invisible at US and not completely depictable with CEUS, the use of virtual navigations systems and fusion imaging have been reported to be a safe, feasible, and effective strategy to perform percutaneous ablations
- Internal markers, such as vessels bifurcations, are the preferred markers to achieve registration and fusion of two imaging modalities.
- The use of CEUS immediately after the treatment during the same session allow for identify incomplete ablations, thus offering the chance of performing a subsequent ablation in the same session
- Percutaneous image-guided ablations have been reported to be feasible for the treatment of adrenal gland metastases
- Protective manoeuver, such as injection of fluid, gas or dedicated gel, has been successfully reported in order to displace surrounding structure from the target of an image-guided ablation
- Microwaves represent one of the most rapidly spreading technique for performing ablation, as it allow to deliver high energy in quite a short time, thus offering the possibility of achieving larger ablation volumes in shorter time in comparison with other techniques, such as radiofrequency.
- The diameter of LA needles is smaller than RFA electrodes and MWA antennas, making LTA safer and more suitable to ablate lesions with at-risk location and/or difficult to be reached
- LTA has also been proposed as the ablation technique of choice in patients with multiple and small tumours variable in size
- Immediate post-procedural CEUS is strongly recommended to assess the completeness of the ablation treatment and to guide the immediate re-treatment in case of residual viable tissue
- Percutaneous image-guided ablation can be proposed as a safe and effective treatment for limited peritoneal metastases.
- Cryoablation has been used for treating successfully a wide variety of tumours.
- PET/CT can be effectively used to guide percutaneous biopsies and ablation.
- A single breath hold period has been reported as sufficient for good visualisation of the target lesion.
- Iceballs formed during cryoablation can be very well detected with CT.
- Cryoablation freezes the tumour and creates an iceball involving the tumour and adjacent tissues; the FDG within tumour remains trapped even after ablation. This helps confirm that the cryoablation iceball, represented by the hypodense zone on CT extends beyond the FDG-avid tumour, thus ensuring adequate coverage of tumour plus a safety margin with cryoablation.

## Conclusions

With the technical improvements of ablative devices, and the increasing evidence of clinical effectiveness of image-guided percutaneous ablations, an increasing number of cases will be referred to the interventional radiologist for a minimally invasive treatment. In this scenario, the number of more complex and unusual cases is also expected to grow. A good knowledge of all the technical options and of some tips and tricks is crucial for performing an effective and safe ablation. Availability of different ablative devices, with the possibility of choosing alterative solutions for image guidance and treatment monitoring, including virtual navigation, fusion imaging and CEUS, could help in the future to further increase the application of minimally invasive ablations to a larger series of patients. Good collaboration with referring physicians and oncologists, and larger dissemination of the knowledge of possible treatments provided by interventional radiologists are crucial for offering a dedicated multidisciplinary and multimodality treatment to oncological patients.
